# Predicting dyscalcemia status in early-lactation multiparous Holstein cows using milk weight and constituent analysis from a single milking at 4 days in milk

**DOI:** 10.3168/jds.2025-26373

**Published:** 2025-07-08

**Authors:** J. A. Seminara, K. R. Callero, S. An, C. M. Salpekar, M. Van Althuis, D. M. Barbano, J. A. A. McArt

**Affiliations:** 1 Department of Population Medicine and Diagnostic Sciences, College of Veterinary Medicine, Cornell University, Ithaca, NY 14853; 2 Southwest New York Dairy, Livestock, and Field Crops Program, Cornell University Cooperative Extension, Cornell University, Ithaca, NY 14853; 3 College of Agriculture and Life Sciences, Cornell University, Ithaca, NY 14853; 4 College of Veterinary Medicine, Cornell University, Ithaca, NY 14853; 5 Department of Food Science, College of Agriculture and Life Sciences, Cornell University, Ithaca, NY 14853

**Keywords:** dyscalcemia, Fourier-transform mid-infrared spectroscopy, maladaptation

## Abstract

Many multiparous cows struggle to adapt to the challenges of the early postpartum period. Dyscalcemia, a condition defined by low blood calcium concentrations at 4 DIM and associated with suboptimal performance across a spectrum of epidemiologically important outcomes (health, productivity, and reproductive success), can be a useful indicator that maladaptive phenotypes are developing in early postpartum dairy cows. Identifying dyscalcemic cows, though theoretically useful from a management perspective, is not logistically viable for commercial dairy farms due to the costs and labor that would be involved in the collection and analysis of samples. Furthermore, timely methods of analysis are lacking. Therefore, our objective in this cross-sectional study was to develop a predictive model for establishing dyscalcemia status by applying machine learning approaches to milk weights and milk constituent data predicted using Fourier-transform mid-infrared spectroscopy (FTIR) from a single milking at 4 DIM. We hypothesized that such a model would have adequate diagnostic characteristics. To test this hypothesis, we collected blood, milk weights, and proportional milk samples from 542 multiparous Holsteins on 5 commercial dairy farms in central New York at 4 DIM. Blood was analyzed for serum total calcium concentration and milk was subjected to FTIR analysis from which constituent data were predicted. Cows were diagnosed as having dyscalcemia if they had serum total calcium concentration ≤2.2 mmol/L at 4 DIM, and as eucalcemic if their serum total calcium concentrations were >2.2 mmol/L at this time. Using milk yield data and the concentrations of anhydrous lactose, true protein, fat, and fatty acid groups, including de novo, mixed, and preformed, all measured in g/100 g milk, as well as milk urea nitrogen (mg/100 g milk), and milk ketone bodies (BHB and acetone; mmol/L) we fit and cross validated random forest models stratified by parity group (2, 3, and ≥4) and farm, for the prediction of dyscalcemia status, our main outcome of interest. We found that on average our models performed favorably with an area under the receiver operating characteristic curve of 0.95 (95% CI: 0.86–1.00), accuracy of 0.90 (95% CI: 0.81–0.98), sensitivity of 0.85 (95% CI: 0.64–1.00), specificity of 0.91 (95% CI: 0.84–1.00), positive predictive value of 0.71 (95% CI: 0.32–1.00) and negative predictive value of 0.96 (95% CI: 0.89–1.00). The data providing the most valuable information to our models were milk weight, and concentrations of lactose and protein. These findings, though limited to a single geographic region, time of day, milking schedule, and season, support the concept that machine learning approaches combined with milk constituent data could become a valuable tool for discriminating between dyscalcemic cows and their eucalcemic counterparts in the early postpartum period.

## INTRODUCTION

At the end of gestation, a dairy cow’s physiological demand for calcium more than doubles due to colostro-genesis and the initiation of lactation (Horst et al., 1997; Megahed et al., 2018). Generated by the sudden onset of calcium secretion into milk, this increase in demand for calcium occurs at a time in the cow’s productive cycle when feed intake is depressed; for most cows these factors combine to cause a drop in blood calcium concentrations during the immediate postpartum period (Ramberg et al., 1984). Though many cows recover blood calcium concentrations within 4 d of parturition, a variable but substantial proportion of cows on commercial dairies (21%–72%) fail to regain eucalcemia by 4 DIM (total serum calcium concentration [**tCa**] >2.2 mmol/L; Mahjoubi et al., 2023; Seminara et al., 2023). This subclinical state of calcium dysregulation is known as dyscalcemia and has been associated with decreases in intake (Seely et al., 2021), rumination time (Seely and McArt, 2024), reproductive performance (Seely and McArt, 2023), and milk production (McArt and Neves, 2020), as well as increases in risk of early-lactation disease (Neves et al., 2018; Seminara et al., 2023) and systemic inflammation (Seminara et al., 2025). Whether or not dyscalcemia is a causal factor driving these negative outcomes is still an active area of research; however, dyscalcemia diagnosis represents a useful indicator that patterns of lactational maladaptation are underway.

Though effective intervention strategies are not well agreed upon, or even well explored in the literature, knowledge of dyscalcemia status at the cow level might be useful information to guide management or treatment decisions for dyscalcemic cows. Despite the theoretical utility of such knowledge, the cost and labor required to employ the gold-standard diagnostic technique (i.e., tCa analysis) for every 4 DIM cow on a commercial dairy makes this approach logistically implausible for most producers. Furthermore, the time it would take for samples to be analyzed at a diagnostic laboratory would likely hamper the producer’s capacity to act on any information gleaned from such analysis. Cost-effective and timely options for the identification of cows adapting poorly to the challenges of lactation are therefore an important prerequisite to the development of strategies that can ameliorate the negative outcomes associated with dyscalcemia in the dairy industry at large.

Fourier-transform mid-infrared (**FTIR**) spectroscopic analysis of milk samples has shown promise in the identification of cows with certain unfavorable health statuses, such as hyperketonemia and mastitis, and has also been used to identify cows slated for herd removal (van der Drift et al., 2012; Bach et al., 2019; Rienesl et al., 2022). This method has not yet been used to predict dyscalcemia status at the individual level in early-lactation cows; however, previous research has associated milk composition and blood calcium status in the early postpartum period, despite some inconsistencies across studies (Chamberlin et al., 2013; Rodrigues et al., 2020; Valldecabres et al., 2024). Moreover, milk constituent concentrations measured by FTIR and yields, most notably of lactose, protein, and de novo fatty acids, differ between cows with different calcium statuses at early postpartum time points (Seminara et al., 2023). This combined evidence is suggestive that there may be a use for FTIR-based analysis in dyscalcemia diagnosis.

Supervised machine learning approaches are an ideal tool for handling the data produced by FTIR-based analyses because they are robust to multicollinearity, a common attribute of this type of data, and functional even when the underlying relationship between the predictors and the outcome is complex or nonlinear (Eskildsen et al., 2016; Jiang et al., 2020; Chan et al., 2022). The exceptional utility of these approaches in spectral applications has already been widely appreciated in the field of dairy science, with models published to predict bovine tuberculosis status, metabolic profiles and milk quality traits, among other outcomes of interest in the industry (Denholm et al., 2020; Frizzarin et al., 2021; Giannuzzi et al., 2022). Giannuzzi et al. (2023) found that these approaches, when applied to FTIR data, were solidly effective for predicting blood concentrations of certain analytes, including serum haptoglobin, at a wide range of DIM; however, the authors were not able to predict calcium concentrations with any reasonable degree of accuracy. In that study, the range of DIM could have been a contributing factor to this difficulty, but it is also possible that calcium concentration itself is simply difficult to ascertain using these methods, even at a more specific time point in lactation. Despite such difficulty, dyscalcemia as a syndrome has properties outside of its calcium perturbances that might be more readily linked to milk constituent composition. As mentioned above, dyscalcemia is associated with changes in feed intake and rumination (Seely et al., 2021; Seely and McArt, 2024), which in turn can both contribute to changes in milk composition (Sutton, 1989; Antanaitis et al., 2024). Furthermore, there is evidence that dyscalcemia may be associated with increased haptoglobin concentrations at 4 DIM, suggesting changes in liver activity that could also contribute to milk compositional changes (Seminara et al., 2025). Therefore, it is possible that the underlying phenotype of maladaptation associated with dyscalcemia might be more readily identifiable than calcium concentration itself using machine learning approaches.

Based on this understanding, our cross-sectional study had a single objective: use common supervised machine learning approaches, applied to estimated milk constituent data, from a single milking at 4 DIM, to develop a predictive model for effectively discriminating between multiparous Holstein cows with and without dyscalcemia at a clinically relevant time point (i.e., 4 DIM, the time of dyscalcemia diagnosis). We hypothesized that our model would perform with acceptable discrimination ability (area under the receiver operating characteristic curve ≥0.70; Swets, 1988; Santini et al., 2021), thereby affirming the potential use of FTIR estimated milk constituent data in the assessment of dyscalcemia at the cow level in early-lactation multiparous Holsteins.

## MATERIALS AND METHODS

The design, analysis, and writing of this cohort-nested cross-sectional study was conducted following both the STROBE-Vet (Strengthening the Reporting of Observational Studies in Epidemiology - Veterinary) and TRIPOD+AI (Transparent Reporting of a multivariable prediction model for Individual Prognosis Or Diagnosis + Artificial Intelligence) reporting guidelines, for use in observational epidemiological studies and in studies developing multivariable prediction models for clinical diagnosis, respectively (Sargeant et al., 2016; Collins et al., 2024).

### Sample Size Calculation

To minimize overfitting in our multivariate predictive models, we calculated our target sample size using equations published in Riley et al. (2019) and using the authors’ recommendations concerning diagnostic models for which previous data were not readily available. Setting the shrinkage factor equal to 0.9, assuming a total of 10 variables would be included in the final models and estimating a conservative Cox-Snell R^2^ value of 0.15, our enrollment goal was 549 multiparous Holsteins. Accounting for a 10% loss due to exclusion criteria, we aimed to enroll 604 cows.

### Study Population

Our study population was housed on 5 commercial dairy farms in central NY, sampled by our laboratory during 3 separate observational prospective cohort studies taking place from July through November 2016 (Bach et al., 2019), June through July 2021 (Seminara et al., 2023), and May through July 2022. Though data from the first 2 prospective cohort designs were repurposed for use in this analysis, the 2022 observational study was designed to assess the main objective of our study, among a few other outcomes that will not be discussed further. Hence, this study is a cross-sectional analysis nested within a set of cohort designs. Farms sampled during those field studies were eligible for inclusion in their respective study if they milked ≥1,000 Holstein cows 3 times daily, had headlocks in the fresh cow pens, used farm management software, housed cows in freestall barns with concrete floors, and fed a negative DCAD diet prepartum. Farm A milked 1,100 cows and averaged 41 kg of milk/d per cow during the study period in 2016. Farm B milked 4,400 cows and averaged 42.3 kg of milk/d per cow during the study period in 2021. Farm C milked 3,100 cows and averaged 39.5 kg of milk/d per cow and farm D milked 1,500 cows and averaged 42.7 kg of milk/d per cow during the study period in 2022. Farm E was sampled twice, in both 2016 and 2022, but none of the same cows were present on the farm at both time points. In 2016, farm E milked 1,060 cows and averaged 43 kg of milk/d per cow and in 2022, farm E milked 1,030 cows and averaged 42.3 kg of milk/d per cow. The research teams did not apply any type of intervention to any of the cows in any of these studies.

### Animal Sampling

Farms A and E were visited twice weekly during the study period in 2016 and all multiparous Holstein cows available for enrollment at 4 DIM were enrolled into the 2016 study on the day that the farm was visited. Farms B, and farms C, D, and E were visited daily during the studies in 2021 and 2022, respectively, and eligible multiparous Holstein cows were enrolled prospectively at 1 DIM. At 4 DIM, milk samples were collected during the second milking of the day between 0930 and 1130 h for all enrolled cows that were present in the fresh cow pen, irrespective of health status. Cows were visually identified from the parlor floor and later verified using parlor software. Proportional milk samplers manufactured by the respective parlor manufacturers were used to collect composite proportional milk samples, continuously incorporating milk from all teats at each stage of the milking. Each sample was manually mixed 3 times after collection before pouring into 60-mL preservative-free plastic sample vials (Aptar CSP Technologies, Auburn, AL). Milk samples were placed in an ice bath at 4°C until analysis. Data concerning production weights of each milking were collected daily from parlor software, for the 4 DIM milking from which samples were collected.

When cows returned from the parlor following the midday milking at 4 DIM, on the same day of and shortly after milk collection, blood samples were collected from coccygeal vessels using 20-ga vacutainer needles into 10-mL evacuated anticoagulant-free tubes (Becton Dickinson, Franklin Lakes, NJ). Blood samples were allowed to clot for at least 30 min at room temperature, serum was separated via centrifugation at 2,000 × *g* for 15 min at 20°C, and 1.5-mL aliquots from each cow were stored at −20°C until analysis.

We enrolled 607 cows into our study at 1 DIM. We excluded cows from the final analysis if a 4 DIM milk sample was not collected (n = 52). Additionally, we excluded cows with previous days carried calf <260 (n = 2), cows that developed milk fever or received i.v. calcium before 4 DIM (n = 4), cows that had invalid spectral data (n = 3), and cows for which milk weight data at that milking were unavailable (n = 4).

### Sample Analysis

Compositional analyses of milk samples were performed by the Barbano Lab in the Department of Food Science at Cornell University (Ithaca, NY) using an FTIR spectrophotometer (Lactoscope model Combi 600, Delta Instruments, Drachten, the Netherlands). These analyses were performed within 24 h for samples collected during the week and between 32 and 56 h of collection for samples collected on the weekend. Optimized basic model filter wavelengths were used in prediction models to determine the content (percent by weight) of milk fat, true protein, and anhydrous lactose (Kaylegian et al., 2009). Calibration reference methods for these models have been previously described (Wojciechowski et al., 2016).

De novo (C4 to C14), mixed (C16, C16:1, and C17) and preformed (≥C18) fatty acids were estimated in g/100 g of milk using partial least squares prediction models (Woolpert et al., 2016). Gas-liquid chromatography reference chemistry was used to calibrate the milk fatty acid parameters, as described by Wojciechowski and Barbano (2016), and the main milk constituents and fatty acids were calibrated using the same 14-sample calibration set. To predict concentrations of milk-predicted blood nonesterified fatty acids, milk BHB, and milk acetone, we used partial least squares models developed by Delta Instruments with parameters 1603, 1601, and 1602, respectively; to predict milk urea nitrogen, we used parameter 502 and calibrated the models using reference chemistry generated by an enzymatic milk urea nitrogen assay (Portnoy et al., 2021).

Serum samples were sent to the New York State Animal Health Diagnostic Center (Ithaca, NY) to be analyzed on an automated analyzer (Hitachi Modular P800, Roche Diagnositcs, Indianapolis, IN) for tCa using commercially available kits (Ca Gen.2, Roche Diagnostics). Analyses from each trial had interassay CV between 1.2% and 1.4% with intra-assay CV between 0.6% and 0.9%.

### Statistical Analysis

RStudio (Posit Software, PBC, Boston, MA) with R version 4.4.0 was used to perform all descriptive calculations and model evaluations.

Cows enrolled in this study were classified as either eucalcemic (tCa >2.2 mmol/L) or dyscalcemic (tCa ≤2.2 mmol/L) based on tCa at 4 DIM (Neves et al., 2018; McArt and Neves, 2020; Seminara et al., 2023).

In an initial screening process, the performances of 5 machine learning algorithms were evaluated for prediction of dyscalcemia from milk constituent and milk weight data at 4 DIM. Algorithms included partial least squares discriminant analysis through the pls package (Liland et al., 2023), support vector machine through the e1071 package (Meyer et al., 2023), lasso and ridge regressions through the glmnet package (Friedman et al., 2010; Tay et al., 2023), and random forest through the randomForest package (Liaw and Wiener, 2002). The 10 predictor variables in each model, prespecified at the time of our sample size calculation, were milk weight, concentrations by weight of lactose, protein, fat, milk urea nitrogen, de novo fatty acids, mixed fatty acids, and preformed fatty acids, and concentrations by volume of milk BHB and milk acetone. The 9 constituent predictors were selected for 3 reasons. First, the biological origin for each compound’s occurrence in milk is different. Second, the predictive equations applied to the FTIR spectra are unique and therefore spectral information would not be excessively redundant. Third, previous research by Seminara et al. (2023) illustrated that at some point in the first week of lactation, each of these constituents differed in the milk of cows with distinct calcium statuses. To screen these algorithms, data were randomly subsampled to create 50 unique pairs of unbalanced training and testing datasets, where within each pair, membership in the training set was mutually exclusive of membership in the testing set. Average performance metrics across all 50 dataset pairs are in Table 1.

Though several of these algorithms performed acceptably, we selected random forest for the final analyses for 3 main reasons. First, unlike many “black box” algorithms, random forest allows the user to ascertain the relative importance of different variables to the models’ accuracy. Second, random forest can be trained to improve discrimination capabilities by specifying data stratification properties useful for naturally stratified data (e.g., parity group and farm; Ye et al., 2013). Lastly, in accordance with previous literature evaluating predictive models for clinical decision making, our preliminary investigations found that random forest had the greatest average accuracy of all the evaluated models, if only marginally (Uddin et al., 2019). The random forest algorithm was then subjected to more rigorous characterization.

In the final analysis, random forest models were evaluated using a balanced dataset, created by combining the original data with synthetic dyscalcemic samples generated for each farm individually, using a synthetic minority over-sampling technique provided in the smotefamily package of R (Siriseriwan, 2024). We chose to balance the dataset because imbalanced data can often reduce the performance of machine learning models (Japkowicz and Stephen, 2002; Guo et al., 2008). This is a common practice in many human health contexts (Weller et al., 2021; Krajnc et al., 2022; Adeoye et al., 2023). Synthetic samples were generated by farm-time in 10 separate simulations, where farm E in 2016 and 2022 were considered to be distinct farm-times. Each simulation produced a dataset balanced by dyscalcemia status with new synthetic minority samples. Each dataset was further broken down into 10 subsets. Nine of these balanced subsets were used to train the random forest, in each instance of cross validation, while the last subset was used as a test subset. Before testing, the minority class of the test subset was randomly down sampled to achieve the same proportion of dyscalcemia as observed in the original data. In each simulation, this process was repeated 10 total times, such that every subset of data served as a testing set for a different random forest model. All models were individually tuned before model fitting using the tuneRF function, within the randomForest package (Liaw and Wiener, 2002), to select the optimal number of candidate variables to randomly sample at each split of the decision tree, or the mtry number, producing the lowest error. Additionally, the strata argument of the random forest function was specified as both farm-time and parity group for all models. The feature importance of each variable, measured as the mean decrease in accuracy of the model when that predictor was removed, was saved from each model to calculate means with 95% CI. Model performance metrics including sensitivity, specificity, positive and negative predictive value, and diagnostic accuracy were generated using the epiR and caret packages (Stevenson and Sergeant, 2024; Kuhn, 2008), based on the predicted test results from each model. Lastly, the pROC package was used to calculate the area under the curve (**AUC**) for a receiver operating characteristic curve based on the model’s predicted probabilities (Robin et al., 2011). The results of this analysis are presented as the mean of each performance metric from all 100 unique random forest models from 10 simulations of 10 cross validations each, with bootstrapped 95% CI.

## RESULTS

### Descriptive Characteristics

Of the 542 multiparous Holsteins included in the final dataset, 433 (80%) were eucalcemic and 109 (20%) were dyscalcemic. Sample size and incidence of dyscalcemia varied among farms. Farm A accounted for 62 cows, 5% of which had dyscalcemia, whereas farm B accounted for 225 cows and had a 22% incidence of dyscalcemia. Farm C contributed 90 cows to the final dataset with 27% of those being dyscalcemic, whereas farm D accounted for 59 cows and had a 12% dyscalcemia incidence. During 2016, our group sampled 51 cows at farm E which, at the time, had a 22% incidence of dyscalcemia. When we returned to farm E in 2022, we collected samples from 55 cows and found that the dyscalcemia risk was 27%.

Within the study population, there were 219 parity 2 cows, 183 parity 3 cows, and 140 parity ≥4 cows. Parity 2 cows had the lowest incidence of dyscalcemia overall at 15%, with parity 3 cows having 22% dyscalcemia, and parity ≥4 cows having the highest incidence at 25%. Among farms, there were numerical differences in the numbers of cows in each parity group, and increasing dyscalcemia incidence with increasing parity did not always hold true. The numbers of cows by parity group across farms and each parity group’s dyscalcemia incidence are reported in Table 2.

Table 3 shows means, 95% CI, and medians between eucalcemic and dyscalcemic cows for tCa measured at 4 DIM, milk weight and milk constituents measured at the midday milking at 4 DIM. Dyscalcemic cows produced less milk and had lower concentrations of lactose and protein with higher concentrations of fat, mixed fatty acids, preformed fatty acids, and acetone than eucalcemic cows. Dyscalcemic cows also had lower tCa than eucalcemic cows, as expected.

### Model Performance and Feature Importance

During cross validation, across 100 iterations of random forest training and subsequent testing on 100 unique pairs of training and testing datasets, random forest models performed favorably in the prediction of dyscalcemia status from milk data at 4 DIM. The mean AUC for all 100 iterations was 0.95 with 95% of model AUC estimates falling between 0.86 to 1.00. The mean for overall accuracy in the prediction of dyscalcemia status across all model iterations was 0.90 with a 95% CI of 0.81 to 0.98. The models had a mean sensitivity of 0.85 and a mean specificity of 0.91, where the 95% CI for these metrics ranged from 0.64 to 1.00 for sensitivity, and from 0.84 to 1.00 for specificity. Positive predictive value among models was highly variable with a mean of 0.71 and 95% of estimates falling between 0.32 and 1.00, whereas negative predictive value was relatively consistent with a mean of 0.96 and 95% CI of 0.89 to 1.00.

**Figure 1.**
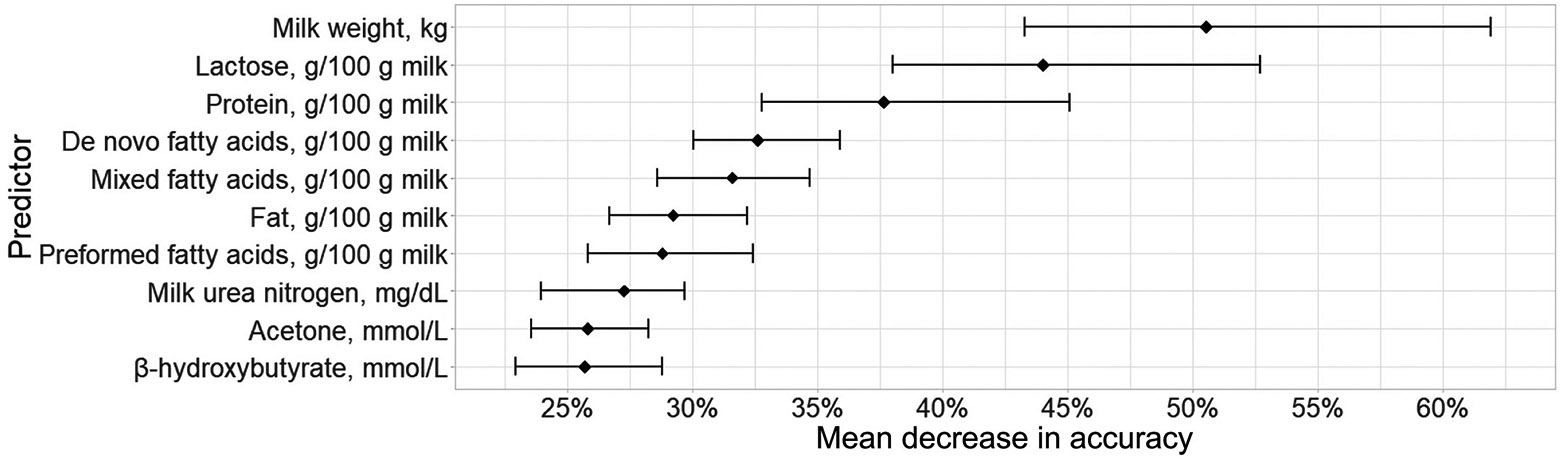
Means and 95% CI for the decreases in prediction accuracy (%) from 100 random forest models, for milk weight and 9 milk constituent predictor variables, used to predict dyscalcemia status (total serum calcium concentration ≤2.2 mmol/L at 4 DIM) in multiparous Holsteins. Milk constituents (lactose, protein, fat, milk urea nitrogen, 3 fatty acid groups [de novo, mixed and preformed] and 2 milk ketone bodies [acetone and BHB]) were estimated using Fourier-transform mid-infrared spectroscopic analysis of milk samples, which were collected, with milk weight data, at the second milking of the day at 4 DIM from 542 multiparous Holsteins on 5 farms in central New York.

The mean decrease in model accuracy (±95% CI) for each of the 10 predictor variables included in our models across all modeling iterations is displayed in Figure 1. The predictors having the greatest effect on model accuracy were milk weight, lactose concentration, and protein concentration, in that order, with the removal of milk weight from the models being responsible for a mean decrease in model accuracy of 51%, lactose concentration for 44%, and protein concentration for 38%. Notably, the 95% CI for each of the top 3 milk characteristics are considerably wider than those of the less informative variables, indicating a high degree of variability in the importance of these variables across model iterations. Although not quite as important to the accuracy of our models, each of the other constituent variables was responsible, individually, for more than a 20% decrease in model accuracy when that variable was removed, confirming that each variable selected provided some useful information to the random forests’ capacity for discriminating dyscalcemic cows from those that were eucalcemic.

## DISCUSSION

Our objective in this cross-sectional study was to develop a machine learning model for discriminating between multiparous Holstein cows with and without dyscalcemia based on milk weight and constituent data that would perform with acceptable diagnostic characteristics. We hypothesized this endeavor would be possible due to underlying differences in the milk constituent profiles of dyscalcemic and eucalcemic cows, and the success of previous researchers in accomplishing similar objectives for other unfavorable health contexts. We found that random forest models consistently displayed more than adequate discrimination abilities, when trained on milk weight and constituent data from a single milking at 4 DIM, at 5 commercial dairy farms in central New York. This finding suggests that machine learning algorithms, if applied to FTIR predicted milk constituents and readily available milk weight data, do have value as a diagnostic tool for discriminating between dyscalcemic and eucalcemic cows in a variety of commercial environments. Leveraging the diagnostic capabilities of this technique could allow producers to fine-tune management or inform therapeutic interventions, which could in turn improve prevention strategies for dyscalcemia or mitigate associated negative outcomes for these maladapted cows.

The metrics of diagnostic utility produced by our models suggest that applying random forest models to milk yield and constituent data at 4 DIM is a potentially viable way to discriminate between cows that may be dyscalcemic and eucalcemic cows. In general, the characteristics of our models favored the correct classification of eucalcemic cows over that of dyscalcemic cows. The high specificity and negative predictive value, 0.91 and 0.96, respectively, indicate that our test was reliable in the identification of eucalcemic cows and produced few false negatives, where a negative result represents a eucalcemia diagnosis. On the opposite side, sensitivity and positive predictive value were relatively low by comparison; the test was able to identify, on average, 85% of all dyscalcemic cows, but only 71% of cows diagnosed as dyscalcemic by our test were actually dyscalcemic. A possible explanation for this finding is that many eucalcemic cows have similar milk constituent profiles to those of dyscalcemic cows. This highlights a limitation to the use of a single time point and diagnostic threshold for all cows: This strategy may not capture all of the biological nuance present in the population. Previous literature illustrates a framework of calcium dynamics that may be more complex than can be described by the simple dyscalcemia/eucalcemia binary, and it may be the case that this added complexity explains similarities between the milk constituent profiles for certain subgroups of dyscalcemic and eucalcemic cows (McArt and Neves, 2020; Seminara et al., 2023). Despite the low positive predictive value of our test, our findings have important practical ramifications. Using this type of algorithm on farms might allow for screening to identify cows that are likely to be healthy while sorting out cows that do need additional monitoring.

In recent years, the combination of milk FTIR analysis and machine learning has become a common approach for the assessment of various outcomes in dairy science (Giannuzzi et al., 2024). Specifically, characterizing the metabolic profiles and identifying the metabolic statuses of cows have received a tremendous amount of attention (Ho et al., 2021; Giannuzzi et al., 2023; Toscano et al., 2023). Although many of these studies have used raw FTIR spectral data in their analyses (Giannuzzi et al., 2023; Walleser et al., 2023; Rovere et al., 2024), many have opted to use FTIR predicted constituents in their modeling as we have done (Heirbaut et al., 2023; Girma et al., 2023; Valldecabres et al., 2024). The latter choice has 2 benefits which are important to note. Most obviously, by translating cryptic FTIR spectra into tangible constituent information, it becomes possible to generate hypotheses about the biological mechanisms underlying the associations observed. Furthermore, it allows the scope of future research to focus on constituents that are potentially more important for understanding maladaptation in early lactation. The second reason relates to the machine learning algorithms we employed. Often, the performance of machine learning algorithms can be improved by a feature selection process, whereby variables of little or redundant importance are removed or condensed into single variables (Pasha and Mohamed, 2020). In the case of FTIR data composed of absorbances across many wavelengths, using partial least squares prediction equations to condense this data into constituent values serves as a form of feature extraction, during which the complex spectral information is largely conserved but the number of predictors in the final model is reduced improving efficiency and performance (Khalid et al., 2014). Future research aimed at establishing which approach, using full spectral data or predicted constituents, is more effective, would be useful for researchers hoping to develop predictive models for dairy cow health.

It is likely that our models’ success was, at least in part, based on the inherent differences in the milk constituent profiles and milking weights of cows with differing calcium status (Seminara et al., 2023). In the majority of our models, the removal of milk weight accounted for the greatest loss in accuracy. Previously published data show that early-lactation milk yields are depressed in dyscalcemic cows, which could be an explanation for our finding (McArt and Neves, 2020; Seminara et al., 2023). Though dyscalcemia cannot be considered a clinical disease event, the repeatable association between dyscalcemia and decreased milk production combined with the knowledge that cows with clinical disease produce less milk than healthy cows, especially around the time of their diagnosis (Rajala-Schultz et al., 1999; Bareille et al., 2003; Dubuc et al., 2011), suggests that dyscalcemic cows may be experiencing some important underlying pathophysiology.

It is difficult to speculate why lactose and protein concentrations were the next most informative features in our models, beyond the numerical differences observed between our groups of interest. These differences in lactose could be related to the liver’s potentially altered gluconeogenic capacity during the acute phase response (White, 2015; Habel and Sundrum, 2020; McCarthy et al., 2020), a process thought to be occurring in dyscalcemic cows (Seminara et al., 2025). The importance of protein in our models could be explained by a different mechanism altogether. Casein proteins, the major protein component of milk, form complexes that carry high concentrations of calcium into milk (Ginger and Grigor, 1999; Neville, 2005), therefore it is reasonable that the protein content of the milk could be related to calcium status. It is also theoretically possible that depressed calcium status in dyscalcemic cows directly influences mammary secretion of both protein and lactose (McManaman and Neville, 2003; Truchet et al., 2014), but the mechanisms linking dyscalcemia to altered milk constituent concentrations remain an area for future research. From a practical perspective, it might be useful that milk weight at a single milking, lactose, and protein were the features accounting for the greatest losses in accuracy when removed from the models, because quantitative data concerning these measures is often readily available to dairy producers through DHI testing and standard parlor software, making more widespread implementation of a model like this potentially viable. Despite this potential for practical utility, our finding that each of the other constituents still accounted for >20% decreases in accuracy when removed from the model individually suggests that a reduced model, trained only on readily available data, would be substantially less effective. Further development of these technologies is therefore warranted to improve the producers’ access to more detailed constituent data before models like those demonstrated herein will be ready for commercial applications.

The design of this study had a few limitations that are important to recognize. First, because effective equations for establishing sample sizes in machine learning prediction contexts are not readily available, our sample size calculation was based on a multivariate logistic regression model. For this reason, it is not entirely clear if or how our sample size would have minimized overfitting in the final models. Second, our study was not designed to assess farm-level variables, although it is likely that these variables were playing a role in determining the dyscalcemia status of the cows in our dataset. With such a wide range of dyscalcemia incidences, we chose to stratify by farm in the final models, effectively accounting for farm-level factors, but this approach does limit the generalizability of our models. It seems likely that if this type of prediction technique were to be used on commercial dairy farms, it might require some degree of calibration to the specific farm. Differences in the underlying relationship between dyscalcemia and milk constituents across farms were difficult to assess with this study design. A third limitation relates to the fact that these samples were all collected in the summer and early fall in New York state, at the midday milking, for herds that milked thrice daily. The relationship between dyscalcemia status and milk constituents across seasons, milking frequencies, times of day, or geographic locations may be different, thus requiring more extensive calibration of models, to account for changes in those parameters. Additionally, we chose to sample cows in the fresh cow pen regardless of their health status which could have created some selection bias, whereby farms with excellent disease detection may have removed cows developing disease to the sick pen by 4 DIM, thus preventing our group from sampling them. Another potential limitation of our study relates to the over-sampling technique that we used to balance the data set. There is some debate as to whether this technique is appropriate due to calibration issues that models may exhibit (van den Goorbergh et al., 2022); however, research across many fields has shown that these techniques improve the accuracy and performance of machine learning models to an extent that likely outweighs problems related to calibration (Weller et al., 2021; Benkendorf et al., 2023; Welvaars et al., 2023).

Overall, though these models are not ready to be used on commercial dairies to identify all healthy or maladapted cows, these methods show great promise as a potential screening tool. As in-line milk measurement of constituents becomes more accessible, it will be useful to have models that can use such information to make predictions about dairy cow health. Expanded sampling of different farm environments, in different geographical areas, in different seasons and at different times of day will help improve this technique and make its widespread application more feasible.

## CONCLUSIONS

Milk weight and constituent data from a single milking at 4 DIM combined with random forest modeling was useful for discriminating between dyscalcemic and eucalcemic cows on multiple farms. Although the models we built were not intended for commercial applications, the approach showed promise as a method of identifying well-adapted eucalcemic cows and similar approaches could become a useful herd-health monitoring tool in the future, either for targeting interventions or fine-tuning upstream management. Understanding the efficacy of these approaches in different farm environments and the degree to which these techniques can affect animal health could be intriguing avenues of further investigation.

## Figures and Tables

**Table 1.** Averages (±95% CI) of performance metrics for 50 simulations of model fitting for partial least squares discriminant analysis (PLS), lasso and ridge regression, support vector machine (SVM) and random forest (RF) models used to predict dyscalcemia status (total serum calcium concentration ≤2.2 mmol/L at 4 DIM) from milk weight and constituent data at 4 DIM, in a cohort of 542 multiparous Holsteins on 5 commercial farms in central NY

	Metric					
Model	Sensitivity	Specificity	Positive predictive value	Negative predictive value	Accuracy	AUC^1^
PLS	0.62 (0.43, 0.80)	0.76 (0.59, 0.89)	0.42 (0.14, 0.77)	0.86 (0.59, 0.98)	0.72 (0.63, 0.81)	0.76 (0.66, 0.83)
Lasso	0.62 (0.43, 0.82)	0.78 (0.59, 0.87)	0.43 (0.16, 0.72)	0.87 (0.60, 0.98)	0.74 (0.64, 0.85)	0.77 (0.66, 0.85)
Ridge	0.60 (0.43, 0.76)	0.79 (0.60, 0.88)	0.44 (0.17, 0.75)	0.86 (0.62, 0.98)	0.75 (0.64, 0.83)	0.77 (0.66, 0.85)
SVM	0.20 (0.00, 0.46)	0.98 (0.94, 1.00)	0.72 (0.30, 1.00)	0.80 (0.48, 0.92)	0.79 (0.50, 0.92)	0.77 (0.64, 0.84)
RF	0.27 (0.12, 0.51)	0.97 (0.92, 1.00)	0.73 (0.27, 1.00)	0.81 (0.53, 0.96)	0.80 (0.58, 0.91)	0.75 (0.62, 0.88)

1 Area under the receiver operating characteristic curve.

**Table 2.** Numbers of cows, by farm and parity, with dyscalcemia incidences of each subgroup in parentheses, for a study population of 542 multiparous Holsteins on 5 commercial farms in central New York^1^

	Parity group, n (dyscalcemia %)		
Farm	2	3	≥4
A	31 (3)	20 (0)	11 (18)
B	91 (16)	68 (25)	66 (26)
C	28 (14)	34 (26)	28 (39)
D	18 (11)	27 (15)	14 (7)
E_2016_	30 (17)	12 (42)	9 (11)
E_2022_	21 (29)	22 (27)	12 (25)

1 Dyscalcemia status was defined as a serum total calcium concentration ≤2.2 mmol/L measured at 4 DIM.

**Table 3.** Descriptive data, including 4 DIM serum tCa concentration and milk weight and FTIR-estimated milk constituent concentrations from a single milking at 4 DIM, in multiparous Holsteins (n = 542) on 5 commercial farms in central New York by dyscalcemia status at 4 DIM (eucalcemic: tCa >2.2 mmol/L; dyscalcemic: tCa ≤2.2 mmol/L)

	Dyscalcemia status			
	
	Eucalcemic		Dyscalcemic	
		
	n = 433		n = 109	
		
Item	Mean (95% CI)	Median	Mean (95% CI)	Median
tCa, mmol/L	2.39 (2.38, 2.40)	2.37	2.08 (2.05, 2.10)	2.12
Milk weight, kg/milking	12.2 (12.0, 12.5)	12.2	10.9 (10.4, 11.4)	11.0
Constituent				
Lactose, g/100 g milk	4.27 (4.26, 4.29)	4.29	4.13 (4.08, 4.18)	4.18
Protein, g/100 g milk	4.13 (4.10, 4.17)	4.10	4.02 (3.93, 4.11)	4.01
Fat, g/100 g milk	5.09 (5.00, 5.17)	4.95	5.63 (5.41, 5.85)	5.58
De novo fatty acids, g/100 g milk	1.10 (1.08, 1.12)	1.07	1.10 (1.05, 1.15)	1.08
Mixed fatty acids, g/100 g milk	1.49 (1.46, 1.52)	1.47	1.62 (1.55, 1.70)	1.62
Preformed fatty acids, g/100 g milk	2.27 (2.21, 2.32)	2.19	2.66 (2.51, 2.81)	2.56
Milk urea nitrogen, mg/100 g milk	11.0 (10.6, 11.3)	11.1	11.6 (10.8, 12.5)	11.1
Acetone, mmol/L	0.09 (0.08, 0.10)	0.09	0.12 (0.10, 0.13)	0.11
BHB, mmol/L	0.06 (0.06, 0.07)	0.06	0.07 (0.06, 0.08)	0.06

## Nonstandard abbreviations used:

AUC: area under the curve
FTIR: Fourier-transform mid-infrared spectroscopy
PLS: partial least squares discriminant analysis
SVM: support vector machine
RF: random forest
tCa: total serum calcium concentration
